# Comparison of Membrane Inlet and Capillary Introduction Miniature Mass Spectrometry for Liquid Analysis

**DOI:** 10.3390/polym11030567

**Published:** 2019-03-26

**Authors:** Wenyan Shi, Xinqiong Lu, Jinbo Zhang, Jianhong Zhao, Lili Yang, Quan Yu, Xiaohao Wang

**Affiliations:** 1 Division of Advanced Manufacturing, Graduate School at Shenzhen, Tsinghua University, Shenzhen 518055, China; shiwenyanu@163.com (W.S.); xqlu@tsinghua.info (X.L.); zhangjb16@mails.tsinghua.edu.cn (J.Z.); jh-zhao17@mails.tsinghua.edu.cn (J.Z.); xhwang@tsinghua.edu.cn (X.W.); 2 State Key Laboratory of Precision Measurement Technology and Instruments, Department of Precision Instrument, Tsinghua University, Beijing 100084, China; 3 Guangdong Province Engineering Research, Center for Urban Water Recycling and Environmental Safety, Graduate School at Shenzhen, Tsinghua University, Shenzhen 518055, China; 4 Hebei Province Key Lab of Measurement Technology and Instrumentation, Yanshan University, Qinhuangdao 066004, China; lily@ysu.net.cn

**Keywords:** membrane inlet, capillary introduction, mass spectrometry, performance comparison

## Abstract

Membrane inlet mass spectrometry (MIMS) is commonly used for detecting the components in liquid samples. When a liquid sample flows through a membrane, certain analytes will permeate into the vacuum chamber of a mass spectrometer from the solution. The properties of the membrane directly determine the substances that can be detected by MIMS. A capillary introduction (CI) method we previously proposed can also be used to analyze gas and volatile organic compounds (VOCs) dissolved in liquids. When CI analysis is carried out, the sample is drawn into the mass spectrometer with no species discrimination. The performance of these two injection methods was compared in this study, and similar response time and limit of detection (LOD) can be acquired. Specifically, MIMS can provide better detection sensitivity for most inorganic gases and volatile organic compounds. In contrast, capillary introduction shows wider compatibility on analyte types and quantitative range, and it requires less sample consumption. As the two injection methods have comparable characteristics and can be coupled with a miniature mass spectrometer, factors such as cost, pollution, device size, and sample consumption should be comprehensively considered when choosing a satisfactory injection method in practical applications.

## 1. Introduction

Mass spectrum (MS) is an analytical method for obtaining chemical composition information by detecting the mass-to-charge ratio of charged particles. It plays an important role in modern analytical science as it has the characteristics of high sensitivity, fast response, and excellent qualitative ability. Sample introduction and ionization are the first stages in the MS analysis process, which determines the types of detectable substance and the complexity of sample pretreatment to some extent [1,2]. There are two sample introduction strategies generally used in conventional mass spectrometers: ionization-then-sampling and introduction-then-ionization. The former is to ionize the sample under atmospheric conditions, followed by ion sampling and delivering to the vacuum for MS detection through a suitable atmospheric interface [3,4]. This method is easy to operate and has wide applicability to nearly all types of samples owing to the flourishing development of atmospheric ionization technology at present. However, relatively complex sampling and transmission systems are required to ensure the efficiency of ion transmission, and large vacuum pumps are generally needed to maintain a high vacuum environment for MS analysis. The latter method is to introduce the sample into the vacuum for ionization, and the generated ions are directly introduced into the analyzer for detection [5]. This strategy shows some advantages like high sample utilization, simple structure, and easy miniaturization, but its application is generally limited to gaseous samples because of the lack of vacuum ionization techniques [6,7].

The detection of gas and volatile organic compounds (VOCs) dissolved in liquids has a wide range of requirements in marine ecology, environment monitoring, and other aspects. For example, determining dissolved nitrogen in seawater can be used to study denitrification, which is helpful in understanding the importance of nitrogen cycle in the marine biogeochemical cycle and ocean–atmosphere exchange [8,9,10]. Moreover, identifying pollutants in water is highly important for environmental monitoring and control [11]. When performing MS analysis of these samples [12,13,14], the introduction-then-ionization strategy is often adopted. In this case, the sample introduction device is critical for balanced injection efficiency and the high-vacuum environment of the instrument. At present, the most commonly used direct introduction techniques are membrane inlet (MI) and capillary introduction (CI) [13], both of which can be used in miniature instruments to achieve high atmospheric sampling efficiency. 

MI uses the selective permeability of a semipermeable membrane for different substances, it is often used with electron impact (EI) ionization sources in current MS systems [5,15,16]. When the sample passes through the membrane, the analyte enters the mass spectra using the principle of pervaporation and is then ionized. This types of MS instrument have been applied to environmental analysis [9,10,11,14,17,18,19,20], online reaction monitoring [15,17,21,22,23], and human body feature detection [23,24,25,26]. By preparing different membranes with multiple permeability characteristics, MIMS can meet various detection requirements [15]. For example, silicone membrane [10], and other polymer membranes are used to detect VOCs and inorganic gas [6] and monitor chemical reaction; meanwhile, zeolite membranes [27] are used to identify isomerides. In comparison, the structure of a CI device is relatively simpler that gaseous substances, along with a considerable amount of air can be directly inhaled from the atmosphere into the mass spectrometer through a capillary [12,28,29]. When analyzing liquid samples, volatile or semivolatile analytes should be extracted from the solution through purging or heating before sampling for CIMS analysis [12,30,31,32]. Our recent experiments have shown that liquid can also be directly introduced into the mass spectrometer by using a capillary with small inner diameter (i.d.), which can effectively reduce ion interferences from the inhaled air during sampling. This introduction strategy has also been successfully used for VOCs detection in liquid samples [28,33] and can be extended to implement vacuum electrospray ionization in miniature ion trap device [34]. The characteristics of both the MIMS and CIMS methods for direct injection of liquid samples is systematically evaluated in this study using a miniature quadrupole mass spectrometer. It will provide a basis for the selection of injection methods to meet the needs of online real-time analysis under different conditions.

## 2. Experimental Instruments and Samples

**Figure 1:**
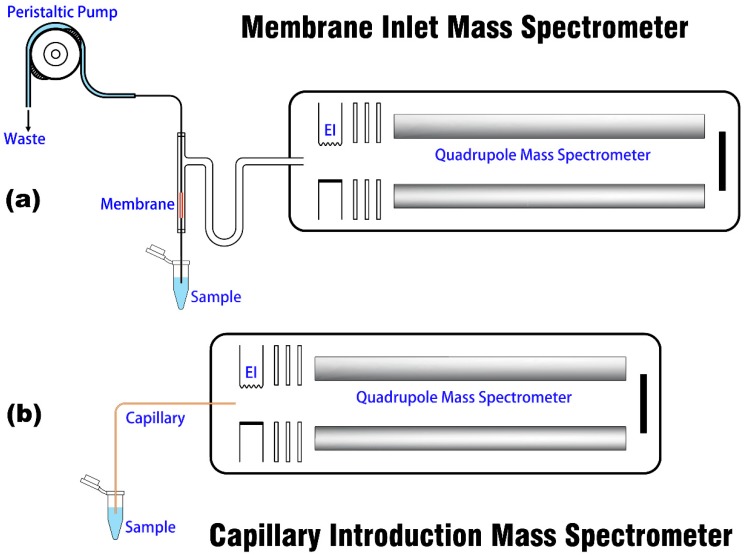
Structural diagrams of (**a**) MIMS and (**b**) CIMS.

Both MI and CI devices are coupled to the same miniature MS platform, and the schematic of the two introduction methods is shown in Figure 1. The mass spectrometer (Prisma 80 QMS-200, Pfeiffer, Asslar, Germany) consists of an EI source, a quadrupole mass analyzer, a vacuum system, a Faraday cup, and an SEM detector, which has been introduced in previous papers [28,35]. The introduction structure of the instrument has been modified to facilitate the change of the sampling device.

The structural diagram of MI is shown in Figure 1a. MI is constructed on the basis of a commercial MI module [10] (Bay Instruments, Easton, OH, USA), which consists of a U-shaped glass tube (i.d. 9 mm) with silicone membrane, a peristaltic pump, a sample introduction tube, and a sample tapping tube. Under the action of the peristaltic pump (BT101L, Lead Fluid, Baoding, China), the liquid sample enters from the inlet and flows through a 25-mm-long membrane tube. When the analyte enters the vacuum environment selectively and is ionized by the ion source, the remaining liquid flows out of the sample tube [36]. The analyte is introduced into the vacuum environment via adsorption, permeation, and desorption under the principle of pervaporation. This process is mainly influenced by many factors, including the membrane dimensions, analyte vapor pressure, temperature, flow velocity and permeability (product of solubility and diffusivity) [15]. In this experiment, the peristaltic pump is connected to the sample tapping tube to avoid the influence of the bubble generated by the squeeze of the peristaltic pump and hose, the permeation of the tube to gas, and other factors on the stability and accuracy of sample detection during sample introduction. For CI analysis, as described in our previous studies [28,29,33], the sampling structure (shown in Figure 1b) is simple that it only requires a capillary with an appropriate i.d. and length to ensure operational vacuum conditions and response time. The liquid sample can be sucked into the mass spectrometer owing to the pressure difference between the surrounding air and the vacuum, and then vaporized and ionized.

Most of the chemicals, such as ethanol (99%), toluene (99%), and 9-methylanthracene (98%) used in this study were purchased from Sinopharm Chemical Reagent Co., Ltd. (Shanghai, China). They are gradually diluted to acquire a series of solutions with different concentrations. All the experiments are conducted at room temperature.

## 3. Results and Discussion

**Figure 2:**
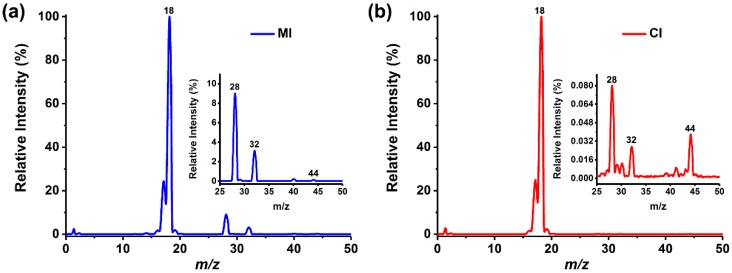
Typical mass spectra of ultrapure water detected by: (**a**) MIMS and (**b**) CIMS. The illustrations are partial enlargements of the mass spectra.

In this study, the characteristics of MI and CI were compared firstly through the detection of ultrapure water, in which a trace amount of air was spontaneously dissolved. MI was driven by a peristaltic pump to flow at a velocity of 1 mL/min. In CI experiment, capillary with an i.d. of 10 μm and a length of approximately 10 cm was selected as the sampling tube, and water was drawn into the MS chamber to form a pressure of 4.9 × 10^−4^ Pa, which was equivalent to 3.7 × 10^−4^ Pa in MI. The pressure of the two introduction methods could ensure the normal operation of the EI source and mass analyzer in the mass spectrometer. The mass spectra acquired by the two introduction modes are shown in Figure 2 showing significant differences. MI separated the water from the dissolved analytes and had different permeability for various substances [36]. As the dissolved N_2_ (*m*/*z* 28), O_2_ (*m*/*z* 32) and other air components had higher permeation rate than water, the ion peaks of corresponding species can be well observed in the spectrum. In contrast, CI directly introduced all substances into the mass spectrometer with no selectivity, hence the spectra were dominated by H_2_O signals.

Sample consumption was a necessary factor to be considered when performing field analysis applications. When VOCs and gases dissolved in liquid samples are measured by MI, the sample consumption is usually several milliliters per minute [10,15,18]. Most samples flow into the inlet tube, pass through the membrane and then flow out from the outlet to become waste. In contrast, the consumption of CI was several nanoliters per minute, which facilitated the detection of trace samples.

The response time of the introduction system, which reflects the efficiency of MS analysis, is an important parameter of online detection. In this study, 0.1% ethanol aqueous solution was used to evaluate the response speed of the two introduction methods. During detection with MI, the sample reached the position of the membrane at approximately 10 s under the action of the peristaltic pump (the flow rate was 1 mL/min). When the sample reached the membrane, permeation happens and the signal of ethanol appeared immediately. The analyte in the liquid was directly transmitted in the form of gas in the vacuum tube with a high diffusion speed after passing through the membrane. Therefore, the time interval between the arrival of the sample to the membrane and the appearance of the signal was extremely short, such that detecting the specific value was difficult. In CI, if the i.d. of the capillary is extremely small, rapid evaporative cooling will occur when the liquid sample enters the vacuum chamber from the atmospheric environment, causing the liquid to freeze at the capillary outlet and hindering continuous detection [34,37,38,39]. Meanwhile, if the i.d. of the capillary tube is extremely large, then the vacuum environment of the mass spectrometer will be seriously damaged when directly feeding the liquid, thereby making the ionization source and detector unable to work properly. In this study, the response time was detected by the capillary tube with an i.d. of 10 μm, an outer diameter of 360 μm, and a length of approximately 10 cm. After the capillary was inserted into the liquid for approximately 10 s, the sample self-aspirated into the vacuum chamber, and the ethanol signal can be observed. It was obvious that regardless of which introduction method was used, the response time detected in the entire MS analysis process was affected by the transmission speed of the solution in the pipeline. The transmission speed of the liquid in the capillary in CI detection was related to the i.d. and length of the capillary, the pressure difference between the two sides of the liquid column, the dynamic viscosity of the sample, and other parameters. Related studies have been conducted to shorten the response time of sample injection to a few seconds, reduce sample consumption to a few nanoliters, and improve sensitivity simultaneously by using pulse capillary introduction (PCI).

**Figure 3:**
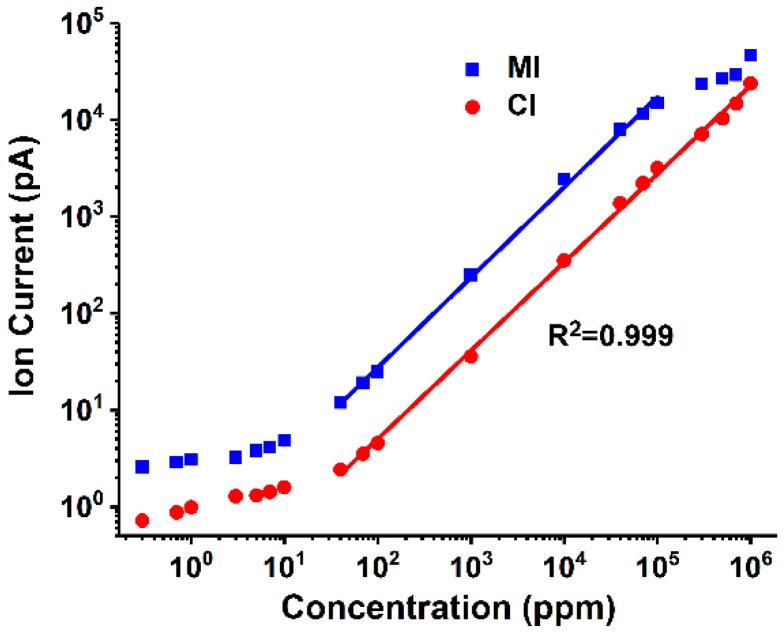
Ion current profiles of ethanol versus sample concentration in MI and CI analysis.

Linear capability is the most important performance index of analysis method. In this study, the linear capability of the two introduction methods was evaluated and compared, and the curve of the ethanol signal (*m*/*z* 31, CH_2_OH^+^) that changes with the sample concentration is shown in Figure 3. The limits of detection (LODs) were calculated using three times the standard deviation of background noise, and the obtained values were 3.4 ppm and 22.8 ppm for the MI and CI analysis, respectively. The fitting curves were also given in Figure 3 with the correlation coefficient (R^2^) being set at 0.999 to represent the linear range of each method. It is found that MI has lower LOD while CI possessed wider linear range. As mentioned previously, MI is selective with better permeability to dissolved gas and VOCs in solution; thus, it can avoid impurities that enter into the vacuum cavity and reduce meaningless collisions between sample components and electrons. Therefore, the signal intensity and sensitivity of MI were better than those of CI. Meanwhile, given that the selectivity of MI was not uniform, the enrichment of dissolved gas in an aqueous solution in a vacuum environment was higher than that of using CI, and its fragment ion peak may interfere with the detection of small molecular organic compounds. Therefore, the background signal of MI for detecting the same ethanol aqueous solution was higher than that of CI.

**Figure 4:**
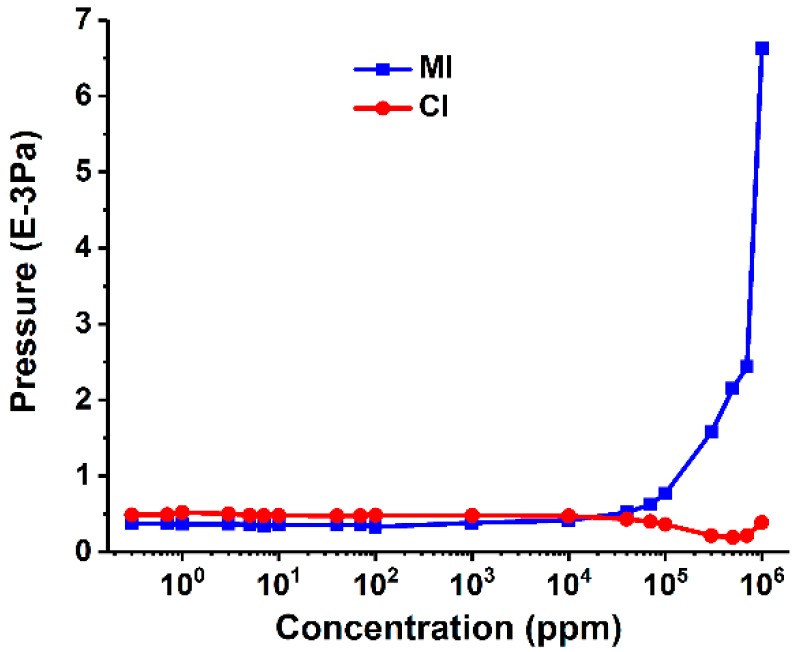
Variation of the chamber pressure with the sample concentration acquired in MI and CI experiments.

When analyzing different solutions, the change of solvent had a significant influence on the introduction characteristics. The variation of pressure with the concentration under different injection methods is shown in Figure 4. The solvent matrix can be considered to be unchanged at a low concentration (approximately <1%), hence no significant pressure difference was observed between different concentrations of the ethanol aqueous solution in MI experiments. With the further increase of ethanol content, the permeability of solution may change, and then more ethanol molecules could penetrate into the vacuum chamber through the silicone membrane, resulting in a significant increase in pressure beyond the range of normal MS operation. Therefore, the application of MI will be limited that it is especially suitable for the analysis of dissolved gases or VOCs in aqueous solutions, but probably not for those samples with organic substances, such as ethanol as solvent [33]. In comparison, the pressure remains relatively stable in CI analysis with the variation of sample concentration. Namely, the sample volume that enters the vacuum chamber does not change remarkably. From this point of view, CI is more compatible with different types of solutions.

**Figure 5:**
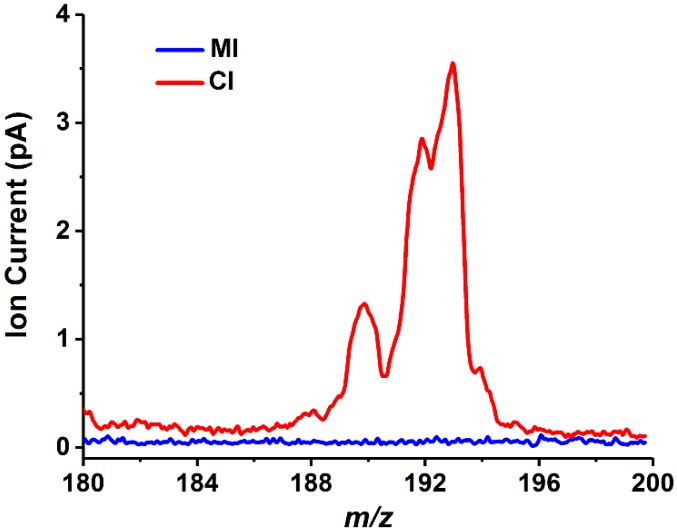
Typical mass spectra of 9-methylanthracene solution acquired in MI and CI experiments.

For now, MI and CI represent different characteristics and are complementary to each other in practical applications. In the MI method, the vapor pressure of the analyte and its solubility and diffusivity in the membrane material determine whether the analyte can be detected by the mass spectrometer, and the diffusivity is relevant with concentration gradient and molecular dimensions [15]. Hence, different membranes may need to be replaced for different samples due to the limited types of analytical substances available for each membrane. In contrast, sample introduction in CI is more direct with no species discrimination that various types of compounds can enter the vacuum environment and be detected by MS. To assess this feature, 9-methylanthracene (boiling point 196 °C −197 °C/12 torr) solution was prepared and detected by MIMS and CIMS in this study, and the obtained mass spectra is shown in Figure 5. As 9-methylanthracene is hardly soluble in water, 5% toluene ethanol was selected as the solvent to form a 300 ppm concentration of 9-methylanthracene solution. When this sample was detected by MIMS, the pressure in the vacuum chamber reaches the level of 0.01 Pa approaching the limit of working pressure of MS. However, the ion signal of 9-methylanthracene can still not be obtained by MI probably because the semivolatile substances cannot pass through the membrane. In comparison, the analyte was detectable by using CI method owing to the straight introduction process. Hence, CI may be more suitable for detecting some semivolatile and poorly water-soluble substances than MI. In addition, it is worth mentioning that the capillary sampler can be transformed into an electrospray ion source only by applying a high voltage on the solution, which enables the analysis of nonvolatile compounds in the solution [34] and can greatly expand the application range of capillary sample introduction. 

## 4. Conclusions

In this study, the direct introduction of liquid samples with MI and CI are compared. MI is a more mature technology and has been widely used. In comparison, CI possesses the merits of simple structure, self- aspiration, ease of operation, and small sample consumption. With regard to analysis performance, the LOD of MI is a little lower than CI when detecting an ethanol aqueous solution. MI is efficient in extracting the analyte from the aqueous solution because most of the membranes have low permeability to water, which can reduces the influence of water on MS and brings higher detection sensitivity to gas and VOCs in solutions. By contrast, CI does not possess species discrimination that it has a wide linear range and can detect more types of substance, especially that part of semivolatile substance detection. The response time of the analysis in both MI and CI at the level of ten seconds, which is mainly affected by the transport process of the samples in their respective injection tubes. Both MI and CI are suitable for miniature mass spectrometer and on-line analysis. When directly analyzing liquid samples using the two introduction methods, each has its own characteristics and can be selected appropriately according to practical needs. In addition, the results of this study can provide guidance for further performance optimization of the CI method, including the development of PCI method mentioned above, which was used to improve the transfer rate of liquid in a capillary. Moreover, when analyzing aqueous solutions, removing water from the samples with high specificity during or after capillary transport is crucial to the sampling efficiency and detection sensitivity for CI, which will make CI more competent as an alternative method of the mature MI.
